# A Case of Congenital Abscence of the Vagina—Artificial Vagina Made by Intestinal Transplantation

**Published:** 1913-12

**Authors:** Thew Wright

**Affiliations:** Attending Surgeon Emergency Hospital; Assistant Surgeon Buffalo General, Erie County, Children’s Hospitals; Buffalo, N. Y.


### A Case of Congenital Abscence of the Vagina—Artificial Vagina Made by Intestinal Transplantation

BY THEW WRIGHT, F. A. C. S.
Attending Surgeon Emergency Hospital
Assistant Surgeon Buffalo General, Erie County, Children's Hospitals
Buffalo, N. Y.

Read Before The Buffalo Academy of Medicine, Jan. 21, 1913

THIS case has been of interest from a psychologic as well as a surgical point of view. The condition with which it deals is fortunately comparatively rare, and it is on that account, perhaps, worthy of mention as well as because it is but the seventh case in the literature in which a section of intestine has been used to form an artificial vagina. When the case was first brought to my attention, and before I had seen and talked with the patient, there was some doubt in my mind as to the propriety of attempting an operation such as I shall describe, but a more careful consideration of the patient's plight made me realize that an attempt to relieve it was not only justifiable but was in fact a duty if one felt that the surgical procedure lay within one's skill. The question of propriety was, of course, whether from a moral standpoint it is proper to render sexual intercourse possible to an unmarried girl by means of an operation, which will not give her the ability to bear children. If we assume that the practice of our profession includes the lessening and elimination of both physical and mental distress, which we should certainly do, then such an operation is not only justifiable but is, I think, demanded in such a case as this.

The desire for sexual gratification is implanted in every sexually developed individual and is nature's means of insuring perpetuation of the species. Each individual may, however, satisfy this desire or not, as he or she wishes. Should an individual choose to abstain from such sexual gratification, such abstinence is, as a rule, harmless, especially when voluntary, many authorities to the contrary notwithstanding. When, however, an individual has the knowledge that although he is apparently sexually developed, the sexual power is absent, the situation is very different. The effect is somewhat different, as a rule, in men and women. In the woman the desire to bear children is the strong point, whereas in man the desire for sexual intercourse is the greater. We must differentiate the michief caused by the inability to procreate and the inability to copulate. Sterility is, as a rule, recognized only after repeated fruitless intercourse.

The single man has no idea that he is sterile unless his suspicion may have been aroused because of some disease that he may have had, such as double epidydimitis. The virgin never has any knowledge of her sterility and always has the power of intercourse, except in such cases as the one under discussion. Knowledge that he is sterile has a detrimental effect upon the man, but this is by no means so marked as that which follows when he is unable to perform the sexual act. The knowledge that he is impotent has often been the cause of melancholia and driven many a man to suicide or to the asylum, even though he was single and his impotence need not have been exposed.

Female sterility is common and it often has a deleterious effect, especially when the husband is anxious for children. Such a condition may cause most pronounced psychic disturbance. Occasionally, to be sure, the knowledge that she can bear no children is looked upon lightly or even with pleasure. Impotence in the male corresponds to the lack of sexual desire in the female. The latter condition is by no means rare and is always a strain upon the married woman. Apart from the fact that intercourse withwithout desire or pleasure becomes more and more distasteful to the wife and makes the married state harder for her; she finds in her lack of sexual appetite something that causes her shame. Such a patient as this is not uncommon and calls for sympathy and tact on the physician's part. Occasionally the knowledge that the ability to become pregnant does not depend upon sexual pleasure will serve to change her feeling and make her marriage duty less irksome.

Now, when we see the mere lack of sexual desire working such havoc, we have no reason to suppose that the rare condition of absolute mechanical inability to perform the sexual act will have any less influence upon the mental state of a fully developed girl than upon a man. It is not the abstinence that causes the feeling of shame and self contempt, but the fact that the abstinence is not of choice but of necessity. The fact that a person in perfect bodily health will choose to undergo an operation of considerable severity and risk is, perhaps, the best evidence of how severe the mental suffering in such a case may be. The case which I shall report is an example, for her decision to submit to an operation was not made of a sudden, under any temporary outburst of sexual feeling, but was the cool decision after months' of consideration, as she had known her condition for a year and had been told of the proposed operation, its limitations and its dangers many times.

The patient, a blond, twenty-two years of age, was to all outward appearances well developed. She had always been healthy and had considered herself normal until a year previous to her coming under my observation, when, owing to the failure of the establishment of menstruation, her mother took her to consult a physician. Although she was at this time 21 years of age, there had never been any sign of menstrual flow, either normal or vicarious, nor of any periodic nervous manifestations.

Her physician discovered her congenital defect at this time and explained the condition to her and to her mother.

Following the appreciation of her defect the patient began to brood over her condition until she became morbid and said she would not live longer unless it could be changed. When her physician brought her to me she was in a serious mental state.

Examination showed the following conditions: A well rounded figure of five feet six inches in height and 135 pounds weight. Breasts well developed. External genitalia perfectly developed and normal in appearance with a normal clitoris. There was an abundance of pubic and axillary hair. With the patient in the lithotomy position, however, examination of the perineum failed to show even the slightest dimple at the site of the normal vaginal orifice. Examination with a finger in the rectum and a sound in the bladder showed that these two organs were in contact. Bimanual examination with a finger in the rectum and hand on the abdomen revealed an oval body about two inches long situated well up on the pelvic brim, which was taken to be an infantile uterus.

The patient was again told the nature of her defect and that it was not in any way life-endangering, and that any attempt to better it would be attended with considerable risk and might be unsuccessful.

With a full understanding of the risks and limitations of the operation she demanded that it be undertaken and underwent it on June 10, 1912.

A careful perusal of the literature showed that the only operations for this condition that could be in any way called successful were those reported by Baldwin, Mori and Mueller in which they had utilized a portion of intestine to form the lining of the newly-made vagina. I chose to follow the plan outlined by Baldwin and successfully carried out by him in four reported cases, and varied its technique but little.

With the patient in the lithotomy position a transverse incision was made in the perineum at the site where the vaginal orfice would normally have been, and through this the bladder and rectum were separated and a roomy channel formed for the reception of the intestinal loop. A long forceps was then placed in this channel and the patient placed in the dorsal position. The abdomen was then opened in the median line and the pelvis inspected. On each side of the pelvis, well up on the brim, was an ovary somewhat larger than the average and a fallopian tube normal in size and appearance. Each tube ended anteriorly in an oval body about one and one-half by one-half inch in size, representing the lateral segments of the uterus. In front both round ligaments were easily demonstratable and they met in the median line in a third ovel body whose presence and nature I am unable to explain, of about the same size as those at the sides. The appearance was as though the uterus had started to develope from three segments instead of two. There was no sign of a vagina. After inspecting the pelvis I selected a portion of the ileum ending about five inches from the ileo-cecal valve as being the loop which could be most readily drawn down to the perineum, and resecting about 12 inches of it, closed the ends of the resected portion with purse string sutures and restored the continuity of the ileum by end to end anastomosis with suture. I then cut the peritoneum of the pelvic floor and placed the center of the resected loop in the jaws of the forceps I had left in the vaginal channel and which an assistant closed and drew down until they were outside the perineal floor. This caused an appreciable traction on the mesentery of the loop, but not enough to interfere with its blood supply. After suturing the pelvic peritoneum around the loop as well as possible, the abdomen was closed in layers and the patient again placed in the lithotomy position. The bowel was then opened at the site of the forceps grasp and the edges of the incision sutured to the vulva and the perineal skin.

The two limbs of the loop of gut were then tightly packed with gauze and the patient returned to her bed. The operation took one hour and forty minutes and was followed by no appreciable shock.

Convalescence was uneventful except for rather prolonged post-operative nausea, which I think was due to the tension on the mesentery of the loop necessary to bring its center down to the perineal skin.

The result in this case has been perfect. Healing was prompt and a careful examination two months after the operation showed a vagina of normal appearance and whose only apparent abnormaliity was the absence of a cervix at the vault and the presence of a slight septum, which later can be removed when desired by clamp pressure.

The result on the mental condition of the patient has been all that could be wished and has fully justified the procedure.

The technique followed in this operation differed from that described by Baldwin only in that suture was employed instead of the Murphy button in making the intestinal anastomosis.

A person communication from Dr. Baldwin tells me that since his last report he has performed the operation successfully several times. I wish here to express my admiration for his ingenuity in devising an operation so excellently adapted to the condition, and one which is attended with so little risk and which can be performed with such assurance of success.

479 Delaware Avenue.

